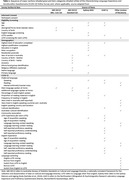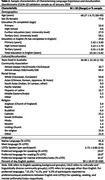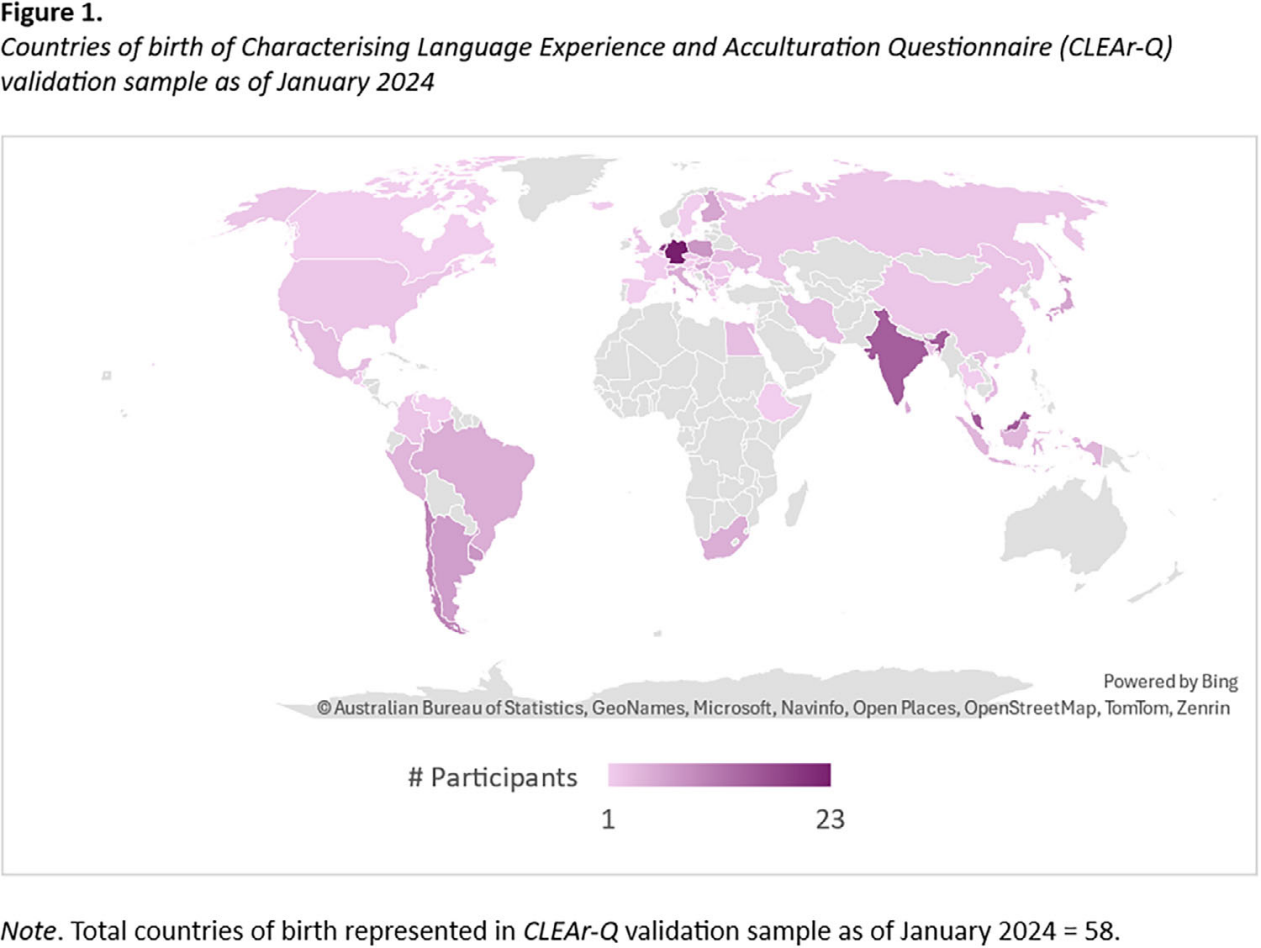# Development and validation of a new measure *Characterising Language Experience and Acculturation (CLEAr‐Q)* to inform the cognitive assessment of culturally and linguistically diverse older adults

**DOI:** 10.1002/alz.090099

**Published:** 2025-01-03

**Authors:** Zara A Page, Karen Croot, Ben C. P. Lam, Henry Brodaty, Nicole A. Kochan

**Affiliations:** ^1^ Centre for Healthy Brain Ageing (CHeBA), UNSW Sydney, Sydney, NSW Australia; ^2^ Centre for Healthy Brain Ageing (CHeBA), University of New South Wales, UNSW Sydney, NSW Australia

## Abstract

**Background:**

Language and cultural factors are known to influence cognitive performance on neuropsychological measures used to assess cognitive impairment and dementia. A new measure, the *Characterising Language Experience and Acculturation Questionnaire (CLEAr‐Q)* was developed to address the gap in access to a brief measure of these factors in the Australian context. The aim is to validate and further develop the *CLEAr‐Q* as a tool to capture linguistic and acculturation variables to improve measurement of cognition in older adults from Culturally and Linguistically Diverse (CALD) backgrounds.

**Methods:**

The *CLEAr‐Q* was initially created as a paper questionnaire and completed by all older adults from CALD backgrounds in the CogSCAN Study. Items were drawn from the literature and adapted based on feedback from the CogSCAN group. An online version for validation with a larger sample was then developed with consultation from a CALD community working group via an adapted participatory research framework. The anonymous survey is completed online in English and data collection will conclude in February 2024. Preliminary analysis (e.g., Pearson correlation) will be conducted to check for redundant items and to select linguistic and acculturation variables (Table 1) to be included in an exploratory factor analysis; this will establish the factor structure and develop a streamlined, psychometrically validated version of the *CLEAr‐Q*.

**Results:**

The CogSCAN sample included 75 participants aged 60‐95 who reported speaking and/or reading a language other than English (LOTE). The validation sample to date consists of 244 participants aged 60‐90 who reported speaking and/or reading a LOTE at a functional level, were born in over 50 overseas countries (Figure 1) and speak on average more than two languages (Table 2). Data analysis is in progress.

**Conclusions:**

Results will contribute to validating the *CLEAr‐Q* and demonstrating its utility to characterise known diversity in CALD samples. Future directions include examining the relative importance of linguistic and acculturation variables from the *CLEAr‐Q* in predicting cognitive performance, which is expected to improve diagnostic accuracy of current neuropsychological measures for assessment of cognitive impairment and dementia in older adults from CALD backgrounds.